# Neuromyelitis optica spectrum disorder in Western China impacts employment and increases financial burden in women

**DOI:** 10.3389/fneur.2022.973163

**Published:** 2022-09-12

**Authors:** Lin Han, Peiwei Hong, Yang Wan, Linjun Cai, Ziyan Shi, Jiancheng Wang, YanLin Lang, Hongyu Zhou

**Affiliations:** ^1^Department of Geriatrics and Neurology, West China School of Public Health and West China Forth Hospital, Sichuan University, Chengdu, China; ^2^West China-PUMC C.C. Chen Institute of Health, Sichuan University, Chengdu, China; ^3^Department of Neurology, West China Hospital, Sichuan University, Chengdu, China

**Keywords:** NMOSD, quality of life, burden of disease, Devic's syndrome, optic neuritis

## Abstract

**Background:**

Neuromyelitis optica spectrum disorder (NMOSD) often leads to disability and exerts a heavy toll on the work and life of affected female patients. This study aimed to analyze the current employment situation and economic burden as well as the risk factors for unemployment in female patients with NMSOD.

**Methods:**

We compared the following unemployment- and employment-related aspects in with NSMOD, which were investigated using questionnaires: the specific impact of NMOSD on work, medical expenses, and factors affecting unemployment.

**Results:**

We enrolled 351 female patients with NMOSD. More than half (54.1%, 190/351) of participants reported that the disease led to unemployment. The unemployment group was significantly older (46.9 ± 12.1 years vs. 39.3 ± 9.4 years, *P* = 0.000), had a higher annual recurrence rate (ARR) (0.6 [inter quartile range [IQR]:0.4–0.9] vs. 0.5 [IQR: 0.3–0.8], *P* = 0.141), and a higher severe disability rate (44.2% vs. 11.2%, *P* = 0.000) than the employment group. Moreover, unemployed patients had lower education levels. The factors influencing unemployment included low education (junior middle-school or below), age, higher ARR, and severe disability (odds ratio [OR] = 6.943, *P* = 0.000; OR = 1.034, *P* = 0.010; OR = 1.778, *P* = 0.038; and OR = 4.972, *P* = 0.000, respectively). Medication and hospitalization costs constituted the principal economic burdens.

**Conclusion:**

The heavy financial burden, employment difficulties, and high unemployment rate are the most prominent concerns of female patients with NMOSD who require more social support and concern.

## Introduction

Neuromyelitis optica spectrum disorder (NMOSD) is an autoimmune disease of the central nervous system, characterized by a high frequency of recurrence and severe disability (1). NMOSD predominantly occurs in young women, up to 7:1 female-to-male ratio, and a high incidence is concentrated at 30–40 years (2, 3). The common clinical manifestations of NMOSD are impaired vision and limb paralysis (3, 4). Numerous patients cannot work because of disability. Concurrently, the high medical expenses exert an extremely heavy burden on patients and families (5, 6). Several studies have reported that NMOSD causes anxiety, fatigue, depression, disability, and deterioration in the quality of life (7, 8). However, compared to extensive investigations of the quality of life and emotions in NMOSD, there is a lack of studies on the employment and economic burden in NMOSD, particularly in women patients. Therefore, this study aimed to estimate the impact of female patients with NMOSD on unemployment and the associated economic burden.

## Methods

### Patients

Questionnaires were sent to patients with NMOSD who were registered in our database between 1 July 2020 and 30 November 2020. Only patients with complete clinical data and those who underwent long-term follow-up were enrolled. The inclusion criteria were as follows: (1) patients with NMOSD who were diagnosed according to the criteria of the International Panel conducted in 2015 (4); (2) those aged between 18 and 65 years, who possessed the ability to work; (3) patients with normal cognitive function, who could answer the questionnaire accurately; (4) and those who provided written informed consent. The exclusion criteria were as follows: (1) myelin oligodendrocyte glycoprotein-antibody positivity; (2) students or retired individuals; (3) patients unable to complete the questionnaire for various reasons such as refusal to accept questionnaires, dishonest responses, contradictory answers, or inability to complete the questionnaire because of severe disability; (4) and absence of complete clinical data; and (5) male patients. The patients in our database were followed up every 6 months face-to-face or by telephonic interview. The patients' medication history, recurrence history, disability level, Expanded Disability Status Scale (EDSS) scores, and disease progression were recorded in the database. EDSS assessment was completed by trained medical specialists. This study was approved by the institutional review board of hospital and all patients provided written, informed consent before participation.

### Study design

This questionnaire survey was divided into three sections: the patient's working conditions and reasons for unemployment; the primary source of income and main areas of expenditure; and the average cost incurred due to the disease (Appendix, p: 1–6). The above-mentioned factors were classified and compared with the clinical data present in the database.

### Statistics

All statistical analyses were performed using SPSS (version 25.0). The data were expressed as mean ± SD or median and inter-quartile range and analyzed using Student's *t*-test or Kruskal–Wallis test to compare the quantitative variables. Meanwhile, data with categorical variables were expressed as numbers and analyzed using the Chi-squared test and binary logistic analysis. *P*-values <0.05 were considered statistically significant.

## Results

We recruited 644 patients with NMOSD for this questionnaire-based study. The questionnaires were completed in the following manner: nine patients completed the questionnaire using a mobile phone application, 91 underwent face-to-face interviews at the outpatient department, and 544 (i.e., the vast majority of the questionnaires) underwent one-on-one inquiry through telephonic interviews.

**Figure 1:**
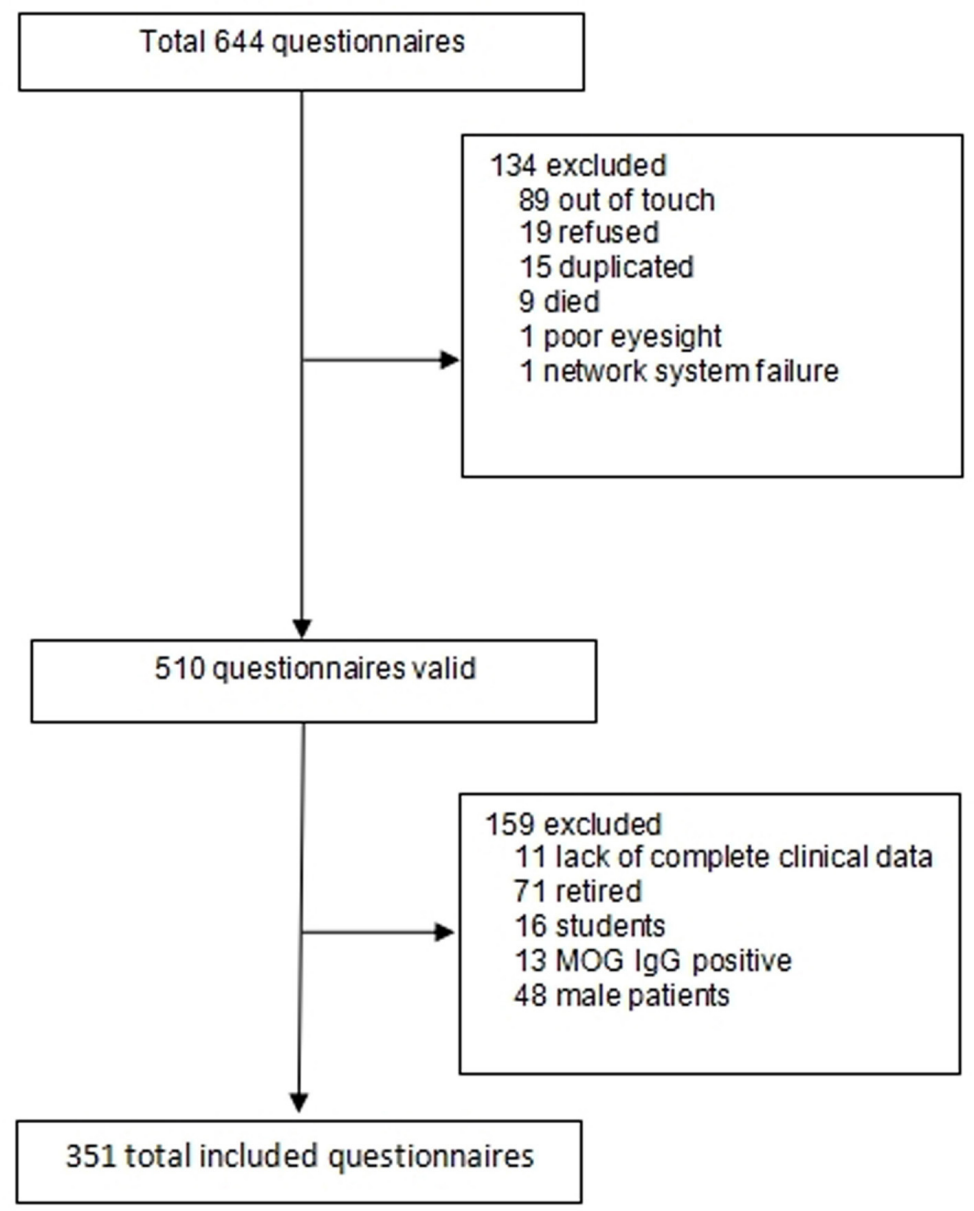
Flowchart of the patient selection process for this questionnaire survey.

A total of 351 of 510 patients with NMOSD who completed the survey fulfilled the inclusion criteria (Figure 1). The average age at the time of the study was 43.4 ± 11.9 years. The average age of the participants in the employment and unemployment groups was 39.3 ± 9.4 years and 46.9 ± 12.1 years, respectively (Table 1). The most common education level in the unemployment group was junior-middle school or lower (76.8%, 146/190), followed by high school (14.2%, 27/190), and bachelor's degree or above (only 8.9%, 17/190). The education level was significantly higher in the employment group than in the unemployment group. The distribution of education levels in the employment group was as follows: junior-middle school or lower (41%, 66/161), high school (15.5%, 25/161), and bachelor's degree or above (43.5%, 70/161) (*P* = 0.000) (Table 1).

**Table 1:** Demographic and clinical data of employed and unemployed patients with female neuromyelitis optica spectrum disorder.

**Characteristics**	**Employed (*n =* 161)**	**Unemployed (*n =* 190)**	***P*-value**
**Age**			
Mean (SD) years	39.3 (9.4)	46.9(12.1)	0.000
**Education**, ***n*** **(%)**			
Junior-middle school or below	66 (41)	146 (76.8)	0.000
High school	25(15.5)	27 (14.2)	
Bachelor's degree or above	70(43.5)	17 (8.9)	
Duration of disease (IQR)	5.3 (2.9–10.4)	5.3 (2.8–8.7)	0.076
Baseline ARR (IQR)	0.5 (0.3–0.8)	0.6 (04.−0.9)	0.141
Number of attacks (IQR)	3.0 (1.0–4.5)	3.0 (2.0–5.0)	0.021
**Baseline EDSS, *n* (%)**			
<4	143 (88.8)	106 (55.8)	0.000
≥4	18 (11.2)	84 (44.2)	
**CNS involvement**, ***n*** **(%)**			0.06
Optic nerve	23(14.3)	20(10.5)	
Spinal cord	55 (34.2)	59 (31.1)	
Coexisting spinal cord and optic nerve abnormalities	77 (47.8)	110(57.9)	
Brian or brainstem	6 (3.7)	1 (0.5)	
**Current treatment, *n* (%)**			
Rituximab	13 (7.3)	8 (4.6)	0.316
Immunosuppressants	135(82.9)	166 (87.4)	
Corticosteroids	129 (78.8)	153 (80.5)	

IQR, inter-quartile range; ARR, Annual Relapse Rate; EDSS, Expanded Disability Status Scale; CNS, central nervous system.

No significant difference was observed with respect to the disease duration between the two groups (5.3, IQR: 2.9–10.4 vs. 5.3, IQR: 2.8–8.7, *P* = 0.076). The median annual recurrence rate (ARR) was no significant difference between the two groups (0.5, IQR: 0.3–0.8 vs. 0.6, IQR: 0.4–0.9, *P* = 0.141) (Table 1). Although the median number of attacks was the same in both groups, the difference was statistically significant (*P* = 0.021). In the employed group, 88.8% of patients had Expanded Disability Status Scale (EDSS) scores <4, and 11.2% of patients had EDSS scores ≥4. EDSS scores <4 were observed in 55.8% of patients in the unemployment group, while 44.2% patients had EDSS scores ≥4. Thus, the EDSS scores showed statistically significant differences between the two groups (*P* = 0.000). Twenty three (14.3%) patients had optic neuritis (ON), 55 (34.2%) had transverse myelitis (TM), and 77 (47.8%) had both TM and ON in the employment group. Twenty (10.5%) patients had ON, 59 (31.1%) had TM, and 110 (57.9%) had both TM and ON in the unemployment group. Overall, no significant differences were observed among these parameters between the two groups (*P* = 0.06). Moreover, the two groups did not differ significantly with respect to the current treatment (*P* = 0.316) (Table 1).

The present study discovered an enormous impact of illness on work. More than half (54.3%, 190/351) of the patients in the study population were unemployed. In the unemployed group, the primary (82.1%, 156/190) reason was the inability to work due to disability, 10% (19/190) reported that it overburdened their colleagues, and 7.9% (15/190) reported other reasons. In the 156 patients, 14.7% (23/156) without physical disability (EDSS <2), 15.4% had mild disability (EDSS = 2 or 2.5), 17.9% (28/156) had moderate disability (EDSS =3 or 3.5), 51.9% (81/156) had moderate disability (EDSS ≥ 4). Furthermore, the disease also has an impact on employed patients. In the employed group, NMOSD necessitated a reduction in the workload in 31.1% (50/161) patients; 11.2% (18/161) reported that it overburdened their colleagues, 8.1% (13/161)took sick leaves frequently, 3.1% (5/161) noted that it was not easy to find a satisfactory job, and 1.9% (3/161) reported discrimination on account of the disease.

**Figure 2:**
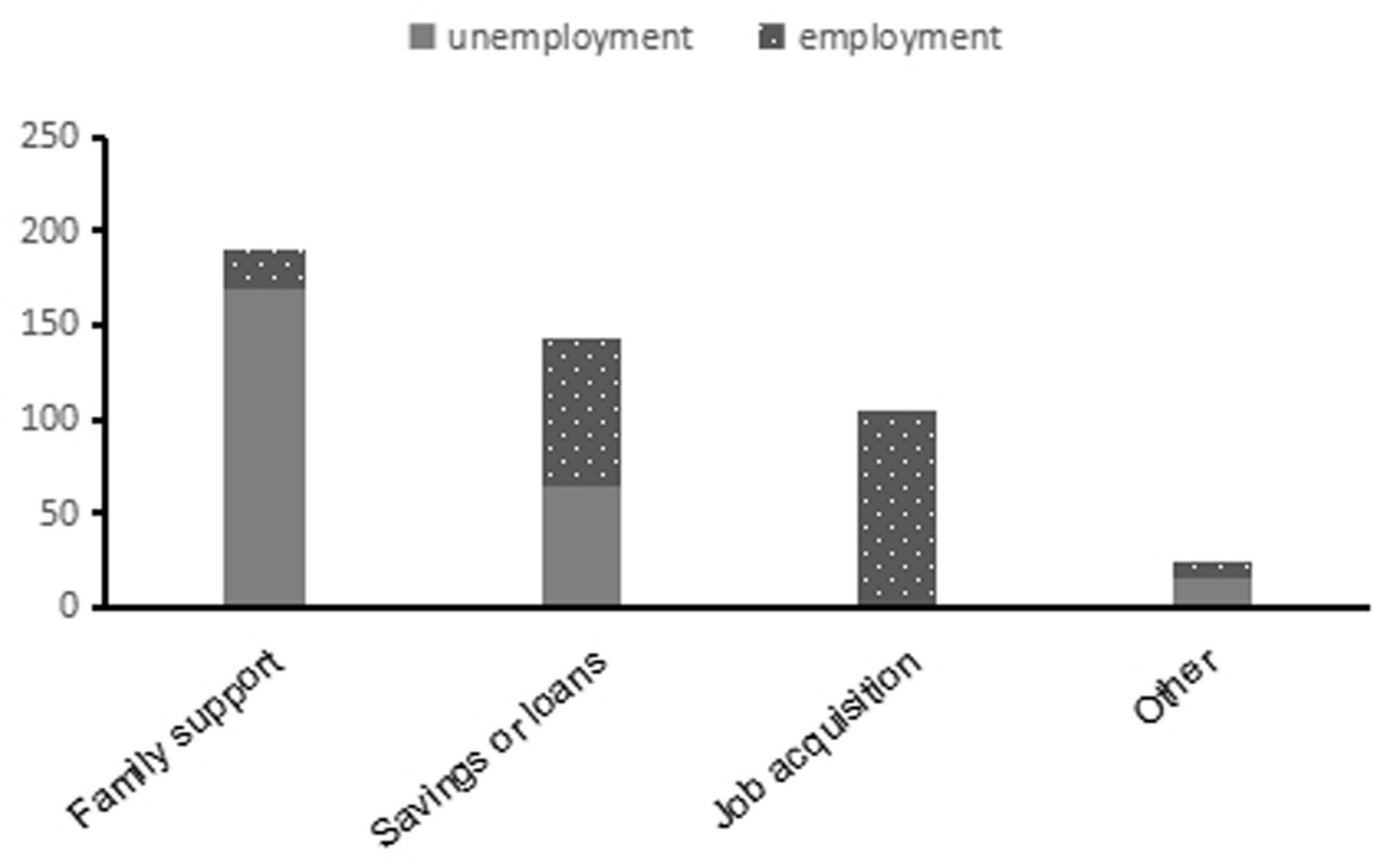
Source of income. More than one source could apply to one participant.

The medical expenses during the remission phase exerted a heavy financial burden on patients with NMOSD, despite excluding the cost of treatment in the acute phase. Long-term medication was administered to 88.3% (310/351) of patients. Most patients chose immunosuppressants (85.8%) and low-dose corticosteroids (80.3%), followed by rituximab (6%). The annual average cost of medication was ¥14,952 compared to ¥600 among patients with minor illnesses living in the same area. Approximately 88.9% (312/351) of patients required repeated hospitalization because of relapse. The annual average cost of hospitalization was ¥29,967 compared to ¥9,088 among their ordinary counterparts [data on the average cost of hospitalization was obtained from the Health Commission of Sichuan Province in 2020 (http://wsjkw.sc.gov.cn)]. Almost all patients (98%, 344/351) underwent regular follow-ups at the outpatient department. The annual average cost of transportation or accommodation was ¥4,716. Expenses related to medication and hospitalization accounted for the greatest proportion of the medical costs (Supplementary Table 1). In our study cohort, the primary source of income was family support (56.4%, 198/351), followed by savings or loans (43.6%, 153/351), earnings from work (35.3%, 124/351), and other sources (6.8%, 24/351). The need for family support was significantly higher in the unemployment group than that in the employment group. However, the proportion of utilization of savings or loans was the opposite, i.e., higher in the employment group than in the unemployment group (Figure 2).

Binary logistic regression analysis showed that the education level, age, ARR, and EDSS score was positively related to unemployment (OR = 6.943, *P* = 0.000; OR = 1.034, *P* = 0.010; OR = 1.778, *P* = 0.038; OR = 4.972, *P* = 0.000), after adjusting for a number of attacks, duration of disease, CNS involvement, and current treatment (Table 2).

**Table 2:** Binary logistic regression analysis showing the influence factors of unemployed.

	***P*-value**	**OR**	**95% CI**
**Education**			
Junior-middle school or below	0.000	6.943	(3.514,13.716)
High school	0.001	4.361	(1.897,10.021)
Bachelor's degree or above		Ref	
EDSS < 4		Ref	
EDSS ≥ 4	0.000	4.972	(2.633,9.217)
Age	0.010	1.034	(1.008,1.060)
ARR	0.038	1.778	(1.034,3.058)

EDSS, Expanded Disability Status Scale; ARR, annual recurrence rate.

## Discussion

Our study found that more than half () of the female patients with NMOSD were unemployed. Most patients lost their jobs due to disability among the unemployed group. The disease continued to impact work even in the employment group, including the need to reduce workload, requiring colleagues to increase their workloads, and taking frequent sick leave. Female patients in the unemployment group were older, less educated, and had a disability of greater severity compared to those in the employment group. Our study also found that female patients with NMOSD had to bear a heavy financial burden. Long-term medications and repeated hospitalizations accounted for the most prominent part of the medical expenses. Since the estimation of the emergency and rehabilitation costs is difficult owing to the influence of the frequency of relapse and disability status, we can infer that the actual real-world medical costs may be significantly higher than those in the remission phase.

A 2019 survey of Chinese patients with NMOSD reported that 90% of unemployment and 100% of school drop-outs were associated with NMOSD (5). NMOSD was the chief cause of unemployment/school drop-out among the patients interviewed. A cross-sectional survey conducted in 25 provinces across China in 2020 reported that the rate of unemployment in patients with NMOSD was 51.9% (109/210) (6), which is in accordance with the results of our study. This finding showed that high rates of unemployment were common and could not be ignored in patients with NMOSD, especially in women.

In this study, binary logistic regression analysis revealed that the lower the degree of education, the higher the EDSS scores, the higher ARR, and the older, which were more relevant to unemployment. It was relatively difficult to find a job with a low education level because of the high replacement rate, which made it more likely for an individual to be replaced after an illness. The same situation also exists in older patients. The development of disability caused by higher ARR and disease was the main cause of unemployment in our study. The more recurrences, the more severe the disability and the easier the unemployment. Furthermore, we analyzed the disability classification of patients. High EDSS scores represent severe disability (9). Among the patients who are unemployed due to diseases, more than half of patients had severe disabilities. Therefore, severe disability had a more detrimental effect on the ability to work. At the same time, we also found that some patients were unemployed even though they had no physical disability. It was inferred that the reason may be related to psychology. Other studies reported that impaired ability to work, heavy economic burden, and physical and emotional stress further contributed to the deterioration in the quality of life (7, 8), and even suicide in some cases (10, 11) It is hoped that increased awareness can bring attention to the plight of NMSOD patients, particularly in women, improve care, and provide more job opportunities.

The heavy economic burden borne by female patients with NMOSD (as observed in this study) is a problem that cannot be ignored. The annual cost of medication in the remission phase was 20 times that of patients with minor illnesses living in the same area, and that of hospitalization in the acute phase was three times that of other patients. Female patients with NMOSD in the United States also face a heavy economic burden (12, 13)). The cost of average hospitalization and outpatient care is as high as $33,954 and $19,325, respectively (14). Patients have to pay out-of-pocket for the most expensive drugs like intravenous immunoglobulin and prescription medicines because of insufficient health insurance coverage (15). Thus, female NMOSD exacts a heavy financial toll, which is a common problem all over the world. However, the difference is that health insurance coverage is wider in foreign countries (16). At present, there is no existing effective treatment for NMOSD, and prevention of relapse is the principal goal of therapy (17, 18). In our study, immunosuppressants and corticosteroids were the two most commonly prescribed drugs (19), and only a small proportion of patients used monoclonal antibodies. This phenomenon may be related to economic reasons and concerns about the safety of new drugs (20–23). Patients choose to delay treatment because of high costs, which eventually leads to repeated relapse and aggravation of disability, further increasing the economic burden. Meanwhile, most unemployed female patients depended on family support, and only a small number relied on savings or loans. We infer that female patients with NMOSD were unemployed due to illness, which further aggravated the economic burden, and finally, the patient had to depend on her family. Thus, families have to shoulder a heavy economical, physical, and emotional burden, which consequently leads to a poor quality of life (24). We opine that more social support and care, including the creation of suitable job opportunities, full health insurance coverage, and strengthening of standard management can ease the burden on NMOSD female patients.

This study had some limitations. There may be regional and survey time differences in this single-center, cross-sectional study and a lack of a control group as well as a comparison with an MS group. The low education level observed in our cohort of patients can also aggravate the unemployment rates. Moreover, not all expenses borne by the patient were accounted for, including the cost of emergency visits, and rehabilitation. Finally, we could not provide the average income in the area and split the data for savings and loans because income in China is private, which means most of the patients did not want to answer these questions.

## Conclusion

In summary, the high unemployment rate, employment difficulties, employment discrimination, and low income were prominent problems faced by NMOSD patients, particularly women. The principal factors affecting employment were the patient's age, education, and disability. Moreover, female NMOSD patients had to bear high medical expenses during outpatient treatment and hospitalization, and thus required attention and support from society, the government, and health insurance policymakers.

## Data availability statement

The raw data supporting the conclusions of this article will be made available by the authors, without undue reservation.

## Ethics statement

The studies involving human participants were reviewed and approved by Biomedical Ethics Sub Committee of West China Hospital of Sichuan University. The patients/participants provided their written informed consent to participate in this study.

## Author contributions

LH: methodology, software, validation, and writing—original draft. YW and PH: conceptualization. JW: visualization and investigation. YL and LC: investigation. ZS: software and validation. HZ: conceptualization and methodology. All authors contributed to the article and approved the submitted version.

## Funding

This work was funded by the National Natural Science Foundation of China (Grant No. 81801202), the Department of Science and Technology of Sichuan Province (Grant No. 2021YFS0173), and 1·3·5 projects for disciplines of excellence–Clinical Research Incubation Project, West China Hospital, Sichuan University (Grant No. 21HXFH041).

## Conflict of interest

The authors declare that the research was conducted in the absence of any commercial or financial relationships that could be construed as a potential conflict of interest.

## Publisher's note

All claims expressed in this article are solely those of the authors and do not necessarily represent those of their affiliated organizations, or those of the publisher, the editors and the reviewers. Any product that may be evaluated in this article, or claim that may be made by its manufacturer, is not guaranteed or endorsed by the publisher.
