# Supplementary material for: Neuromyelitis optica spectrum disorder in Western China impacts employment and increases financial burden in women
Source: Front Neurol. 2022 Sep 12;13:973163. doi: 10.3389/fneur.2022.973163 (PMC9510986; doi:10.3389/fneur.2022.973163)
Supplement: Supplementary file 1 [file Table_1.docx]

| Average annual cost | Number | Range (¥) | Average value (¥) | Comparison value ((¥) |
| --- | --- | --- | --- | --- |
| Care workers | 7 | 19,200-66,000 | 40452 |  |
| Medication | 310 | 120-120,000 | 14,952 | 600 |
| Transportation or accommodation | 344 | 60-38,400 | 4716 |  |
| Hospitalization expenses | 312 | 4000-160,000 | 29,967 | 9088 |
| Lost wages | 8 | 150-5000 | 1856 |  |

Table 1
